# Two new species and one new record of the genus *Tylopilus* (Boletaceae) from Indian Himalaya with morphological details and phylogenetic estimations

**DOI:** 10.3897/mycokeys.33.23703

**Published:** 2018-04-13

**Authors:** Dyutiparna Chakraborty, Alfredo Vizzini, Kanad Das

**Affiliations:** 1 Botanical Survey of India, Cryptogamic Unit, P.O. Botanic Garden, Howrah – 711103, India; 2 Department of Life Sciences and Systems Biology, University of Torino, Viale P.A. Mattioli 25, I-10125 Torino, Italy

**Keywords:** Agaricomycetes, Basidiomycota, Boletales, macrofungi, phylogeny, Sikkim, taxonomy

## Abstract

*Tylopilus himalayanus* and *T. pseudoballoui* are described as new species from two Himalayan states (Sikkim and Uttarakhand) in India. *Tylopilus himalayanus* is characterised by a unique combination of features: reddish- or brownish-grey to purplish-grey then brown to reddish-brown or darker pileus, absence of olive or violet tinges on stipe surface, angular pores, stipe without reticulum or rarely with a faint reticulum restricted to the very apex, bitter taste of the context and positive macrochemical colour reaction of the stipe context with KOH (dark orange) and FeSO_4_ (dark green), medium sized (10.9–14.4 × 3.9–4.9 µm) basidiospores and occurrence under coniferous trees; *T. pseudoballoui* is distinguished by orange-yellow to brown-yellow sticky pileus, pale yellow pore surface with pinkish hues that turns pale to greyish-orange on bruising; angular pores, stipe concolorous to pileus with pruinose but never reticulate surface, ixocutis pattern of pileipellis and occurrence under broadleaf trees. Another species, *T. neofelleus*, which was reported earlier from China and Japan, was also collected from Sikkim and reported for the first time from India. All three species are described with morphological details and two-locus based (nrLSU and nrITS) phylogenetic data.

## Introduction

The genus *Tylopilus* P. Karst., one of the less attractive to eye-catching ectomycorrhizal taxa (associated mainly with Fagales and Pinaceae) in the family Boletaceae, is featured by its dry, glabrous to subvelvety pileus, white to greyish pore surface usually becoming flesh pink to purple-brown at maturity, immutable to slightly brownish or becoming blue-green context on bruising, solid stipe with pruina or reticulation over the surface, absence of annulus or veil, flesh-pink to dull flesh-ochre spore print, smooth pink-coloured basidiospores, presence of pleurocystidia and absence of clamp-connections (Smith and Thiers 1971, Wolfe 1979, Singer 1986, Wu et al. 2014). Further, this genus was divided into two subgenera namely, T. subg. Tylopilus and T. subg. Porphyrellus (Smith and Thiers 1971, Singer 1975). The former subgenus is characterised by “spores with pale cinnamon-yellow to pale yellow walls in KOH and IKI; context usually unchanging or rust coloured on injury; context not turning red-brown in KOH”, whereas the latter is characterised by “spores with dark brown walls in KOH and IKI; context usually turning blue-green on injury then becoming red-brown and, in some taxa, the context becoming red-brown in KOH” (Wolfe 1979). From all over the world (mostly from North America, Australia, Asia, Africa and Europe), about 75 species are reported (Kirk et al. 2008, Magnago et al. 2017). Like some other morphology-based genera in Boletaceae, the traditional concept of *Tylopilus* (*Tylopilus* s.l.) was split and has given birth to a few other genera with the recent advancement of multi-locus phylogeny. *Tylopilus* s.l. appeared as polyphyletic and evolved in 11 different lineages during the course of evolution (Nuhn et al. 2013, Wu et al. 2014). Thus, taxonomic placement of the members of this genus are still floating and many previously considered *Tylopilus* species are shifted into new genera such as *Zangia* Yan C. Li & Zhu L. Yang, *Australopilus* Halling & Fechner and *Harrya* Halling, Nuhn & Osmundson (Li et al. 2011, Halling et al. 2012). According to Wu et al. (2014), all the 11 clades consisting of the members of *Tylopilus* come under five subfamilies (Austroboletoideae, Leccinoideae, Boletoideae, *Pulveroboletus* group and Zangioideae) in Boletaceae and *Tylopilus* s.s., typified by *Tylopilus felleus* (Bull.) P. Karst., is placed within the subfamily Boletoideae.

The entire Indian Himalayan region comes under one (“Himalaya”) of the globally acclaimed biodiversity hotspots and thus has immensely diverse mycobiota (including macrofungi) apart from its myriad flora and fauna. A wide range of phytogeographic variations with the presence of large numbers of ectomycorrhizal host plants, cold to warm monsoon, favourable humidity and plenty of rainfall, supports the growth of ectomycorrhizal mushrooms of Boletaceae. However, due to the lack of mushroom-explorers or mushroom-taxonomists, most of the areas of Indian Himalaya remain unexplored in terms of Boletaceae (only 80 species belonging to 23 genera, while more than 1050 species from 66 genera are reported from the world) (Chakraborty et al. 2017). During macrofungal surveys to different forested areas of Eastern Himalaya (Sikkim) and Western Himalaya, three interesting members of *Tylopilus* were collected separately. Detailed macro- and micromorphological studies followed by phylogenetic analyses based on nrLSU and nrITS sequences, confirm the novelty of two of them and are proposed here as *T. himalayanus* and *T. pseudoballoui*, whereas the third one appeared as conspecific to *T. neofelleus* (a species so far reported from Japan and China, Gelardi et al. 2015) and is reported as a new record for Indian mycobiota.

## Materials and methods

### Morphological study

Macromorphological characters and habitat details were noted from fresh, young to mature basidiomata in the field and in base-camp. After recording the macromorphological characters, basidiomata were dried with a field drier. Photographs of these fresh and dry basidiomata and microphotographs were taken with the aid of Canon Power Shot SX 50HS, Canon SX 220 HS and Nikon-DS-Ri1 (dedicated to Nikon Eclipse N*i* compound microscope) cameras. Colour codes and terms are mostly from Methuen Handbook of Colour (Kornerup and Wanscher, 1978). Micromorphological characters were observed with compound microscopes (Nikon Eclipse N*i*-U and Olympus CX 41). Sections from dry specimens were mounted in a mixture of 5% KOH, 1% Phloxine and 1% Congo red or in distilled water. Micromorphological drawings were prepared with a drawing tube (attached to the Nikon Eclipse N*i* microscope) at 1000×. The basidium length excludes that of the sterigmata. Basidiospore measurements were recorded in profile view from 30 basidiospores. Spore measurements and length/width ratios (Q) are recorded here as: minimum–**mean**–maximum. Herbarium codes follow Thiers (continuously updated).

### DNA extraction, polymerase chain reaction (PCR) and sequencing

Genomic DNA (for all the species) was extracted from 100 mg of dry basidiomata using the InstaGeneTM Matrix Genomic DNA isolation kit (Biorad, USA) following the manufacturer’s instructions. PCR amplification primers were ITS1 and ITS4 (nrITS region) and LR0R and LR7 (nrLSU region) (White et al. 1990). PCR amplification on “ABI Veriti” thermal cycler protocols for nrITS and nrLSU regions were after Das et al. (2017). The PCR products were then purified using the QIAquick PCR Purification Kit (QIAGEN, Germany) before they were sent for sequencing. Both strands of the PCR fragments were sequenced on the 3730xl DNA Analyzer (Applied Biosystems, USA) using the amplifying primers and assembled using Sequencer (Gene Codes Corporation, USA). The nrITS and nrLSU sequences for DC 16-64 (MG777524 and MG777529), DC 16-63 (MG777523 and MG777525), DC 17-31 (MG799323 and MG799326), DC 17-25 (MG799322 and MG799328), DC 17-30 (MG799329 and MG799327) and DC 17-35 (MG799324 and MG799325), respectively, were deposited in GenBank.

### Phylogenetic analyses

The nrLSU and nrITS datasets were assembled according to recent previous studies on this genus (Gelardi et al. 2015, Magnago et al. 2017) and from BLAST (Altschul et al. 1997) searches in GenBank (Clark et al. 2016). As most *Tylopilus* collections in GenBank are not provided with both molecular markers, we were unable to establish a combined nrITS+nrLSU dataset and so have opted for present separate nrLSU and nrITS phylogenetic inferences. These (nrITS and nrLSU) sequences were aligned separately in MAFFT 7.305 (Katoh and Standley 2013). For the nrLSU dataset, *Xanthoconium sinense* (KT990666 and KT990664) and *X. purpureum* (KT990663) from Boletaceae were used as outgroup taxa. Similarly, for the nrITS dataset, two sequences from *Gyroporus* (KX869874, GQ166901), another genus in Boletales (Gyroporaceae), were used as the outgroup. Phylip file formats were created in AliView (Larsson 2014) using default settings. Phylogenies were reconstructed using Maximum Likelihood (ML) in RAxML 7.2.6 (Stamatakis 2006) in GTRGAMMA substitution model. All parameters in the ML analyses used the default settings in RAxML and Maximum Likelihood bootstrap percentage (MLB) were obtained using nonparametric bootstrapping with 1000 replicates. Additionally (to generate supplementary data), nrLSU and nrITS sequences were also phylogenetically analysed using Bayesian analysis. The best-fit models of nucleotide evolution for nrLSU and nrITS datasets (TIMef and TrNef+G, respectively) were obtained in MrModeltest 3.7 (Posada and Crandall 1998). Bayesian inferences were computed independently twice in MrBayes v.3.2.2 (Ronquist et al. 2012), under TIMef (for nrLSU) and TrNef+G (for nrITS) models, respectively. Bayesian posterior probabilities values (BPP) were calculated in two simultaneous runs with the Markov Chain Monte Carlo (MCMC) algorithm (Larget and Simon 1999). Markov chains were run for 1000000 generations, saving a tree every 100th generation. These analyses were terminated when the average standard deviation of split frequencies fell below 0.01. The first 25% of trees was discarded as burn-in (Hall 2004). The convergence of runs was visually assessed using Trace function in Tracer version 1.6.0 (Rambaut et al. 2013).

## Results

### Phylogenetic inferences

**Figure 1.**
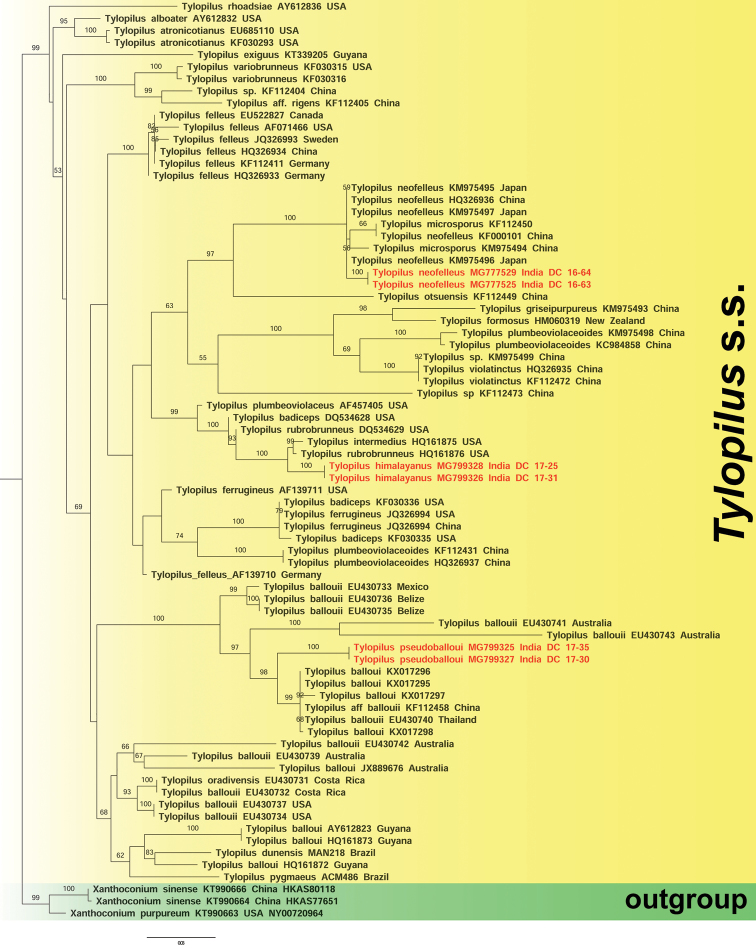
nrLSU based phylogram generated from Maximum likelihood (ML) analysis under GTRGAMMA substitution model depicting the placement of *Tylopilus neofelleus, T. pseudoballoui* and *T. himalayanus* within *Tylopilus* s.s. Two species of *Xanthoconium* (*X. sinense* and *X. purpureum*) were used as outgroup taxa. ML Bootstrap percentage (MLB) derived from this analysis (MLB >50%) are shown above or beneath the branches. Two novel species and a new record for Indian mycobiota are highlighted in bold and red font. GenBank accession no. and country name (when available) for each species are shown after the species name.

**Figure 2.**
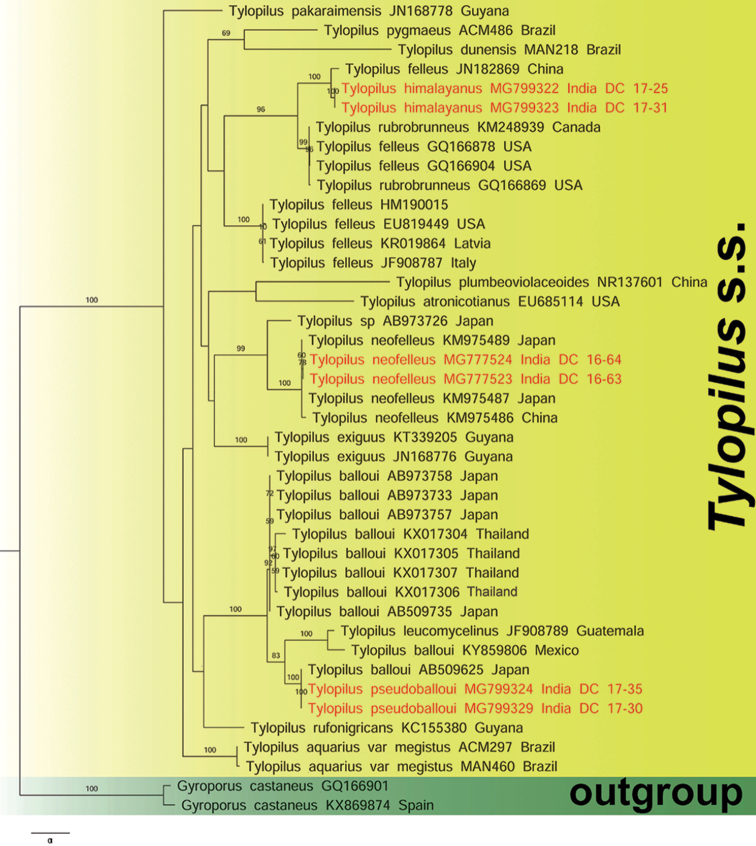
nrITS based phylogram generated from Maximum Likelihood (ML) analysis under GTRGAMMA substitution model depicting the placement of *Tylopilus neofelleus, T. pseudoballoui* and *T. himalayanus* within *Tylopilus* s.s. Two sequences of *Gyroporus castaneus* were used as outgroup. ML Bootstrap percentage (MLB) derived from this analysis (MLB >50%) are shown above or beneath the branches. Two novel species and a new record for Indian mycobiota are highlighted in bold and red font. GenBank accession no. and country name (when available) for each species are shown after the species name.

The nrLSU- and nrITS-based phylogenetic analyses (Figs 1–2 and Suppl. materials 1–2) consist of 76 and 42 sequences, respectively. In our nrLSU based ML and BI analyses (Figs 1 and Suppl. material 1, respectively), the two Indian collections of *T. himalayanus* (DC 17–25 and DC 17–31) clustered together and appeared sister (MLB = 100%, BPP = 1) to the North American *T. intermedius* (HQ161875) and *T. rubrobrunneus* (HQ161875). However, our species with its two sequences (MG799328 and MG799326) is recovered as a distinct species. In these same analyses, the two Indian specimens of *T. pseudoballoui* (DC 17–30 and DC 17–35) are sister (MLB = 98%, BPP = 1) to a strongly supported clade (MLB = 99%, BPP = 1) formed by six sequences named as “*T. balloui*” or “T. aff. balloui” (EU430740, KX017298, KF112458, KX017295, KX017296, KX017297) from Asia. However, our Indian collections (MG799325 and MG799327) are recovered as a distinct species. Our other two Indian collections of *T. neofelleus* (DC 16–45 and DC 16–63) clustered along with all the Asian counterparts (KM975495, HQ326936, KM975496, KM975497, KM975494, KF000101, KF112450) in a strongly supported clade (MLB = 100%, BPP = 1), indicating its conspecificity.

Similarly, in our nrITS-based ML and BI analyses (Figs 2 and Suppl. material 2, respectively), the two Indian collections of *T. himalayanus* (DC 17–25 and DC 17–31), along with a collection of China (JN182869, wrongly labelled as “*Tylopilus felleus*”), appeared sister (MLB = 96%), in the ML analysis, or close, in the BI analysis, to a clade consisting of two *T. rubrobrunneus* sequences (KM248939 from Canada, GQ166869 from USA) and two “*T. felleus*” from USA (GQ166878, GQ166904). However, our collection is recovered as a separate species. The two Indian specimens of *T. pseudoballoui* (DC 17-30 and DC 17-35) clustered strongly (MLB = 100%, BPP = 1) with a Japanese sequence of “*T. balloui*” (AB509625) and appeared as sister (MLB = 83%), in the ML analysis, to a clade consisting of one Mexican collection (represented by KY859806 and labelled as “*Tylopilus ballouii*”) and *Tylopilus leucomycelinus* (JF908789) from Guatemala and as sister (BPP = 0.85) whereas, in the BI analysis, to a clade (MLB = 100%) formed by eight Asian sequences of “*T. balloui*”, four from Japan (AB973733, AB973757, AB973758, AB973735) and four from Thailand (KX017304, KX017306, KX017305, KX017307). However, our species is recovered as a distinct species. Finally, as in the nrLSU analysis, here also the two Indian collections of *T. neofelleus* are strongly clustered (MLB = 100%, BPP = 1) with three Asian counterparts (KM975487 and KM975489 from Japan, KM975486 from China), showing their conspecificity.

### Taxonomy

#### Tylopilus himalayanus

Taxon classificationFungiBoletalesBoletaceae

D. Chakr., K. Das & Vizzini sp. nov.

MB823975

Figs 3 , 4

##### Holotype.

India. Sikkim: East District, Upper Chandmari, 1977 m alt., N27°23'13.7", E88°46'42.9", 26 Aug 2017, *D. Chakraborty & K. Das*, DC 17-25 (CAL 1649).

##### Diagnosis.

Distinct from all allied taxa by a combination of sequence data (nrITS and nrLSU), reddish- or brownish-grey to purplish-grey, then brown to reddish-brown pileus in basidiomata, absence of olive or violet tinges on stipe surface, presence of angular pores, stipe without reticulum or rarely with a faint reticulum restricted to the very apex, bitter taste of the context, positive reaction of the stipe context with KOH (dark orange) and FeSO_4_ (dark green) and medium sized (10.9–14.4 × 3.9–4.9 µm) basidiospores.

##### Etymology.

Referring to Indian Himalaya, the type locality.

##### Description.

Pileus 71–120 mm diam., initially convex then plano-convex to applanate, surface dry, matte to somewhat subvelvety, faintly areolate, brownish-grey, dull red, reddish-grey to purplish-grey or greyish-magenta (11–13B–C2–3) when young, gradually brown to reddish-brown (7E4–9D4) or darker, paler greyish-yellow (4C4) towards margin, pale yellow (2A3) at margin; margin entire, decurved to plane with a narrow flap of tissue, blond (4C4). Pore surface greyish-yellow (3C4) when young, pinkish-white (8A2) with age, turning greyish-brown (6D3) on bruising; pores angular, 2/mm. Tubes adnexed to subdecurrent, 5–6 mm long, whitish-brown to brownish, light brown to brown (26B2–3) on bruising. Stipe 95–155 × 20–32 mm, mostly subclavate, hollow, pale yellow (1–2A3) at apex, brownish towards base but never violaceous; surface usually without any reticulum, but sometimes faintly reticulate at apex (1/8^th^ from the juncture), the rest longitudinally striate. Context up to 16 mm thick in pileus, milk white (1A2), unchanging when exposed. Stipe context turning dark green with FeSO_4_, dark orange with 5% KOH, orange with 10% NH_4_OH. Taste bitter. Spore print not obtained.

Basidiospores 10.9–**12.5**–14.4 × 3.9–**4.5**–4.9 µm, (n = 30; Q = 2.51–**2.75**–3.25), elongated to fusiform, inequilateral, thin-walled, smooth under light microscope. Basidia 30–40 × 9–10 µm, four-spored, clavate. Pleurocystidia 27–54 × 8–10.5 µm, emergent up to 30 µm, fusoid to ventricose, appendiculate. Tube edge sterile; cheilocystidia 32–48 × 5.2–8 µm, common, clavate to cylindrical, subfusoid to ventricose. Hymenophoral trama divergent, hyphae septate, gelatinous, up to 6 µm wide. Pileipellis a trichoderm, up to 150 µm thick, composed of erect hyphae, somewhat interwoven, encrusted, brown pigmented; terminal elements 20–50 × 5–10 µm, cylindrical to subcylindrical, sometimes subfusoid, content brown pigmented. Stipitipellis a cutis, made up of sub-parallel repent hyphae; caulocystidia not observed; caulobasidia similar to that of hymenial basidia.

**Figure 3.**
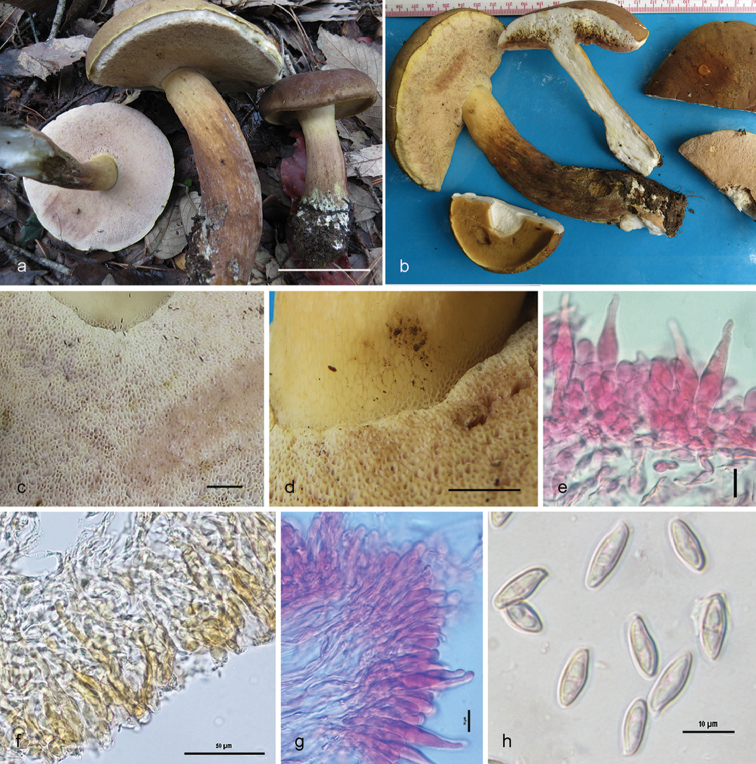
*Tylopilus himalayanus* (DC 17-25, holotype). **a, b** Fresh basidiomata in the field and in basecamp **c** Pore surface after bruising **d** Surface of stipe apex with reticulation **e** Pleurocystidia **f** Pileipellis **g** Tube edge **h** Basidiospores. Scale bars: 50 μm (**f**); 10 μm (**e, g, h**); 5 mm (**c, d**); 5 cm (**a**).

**Figure 4.**
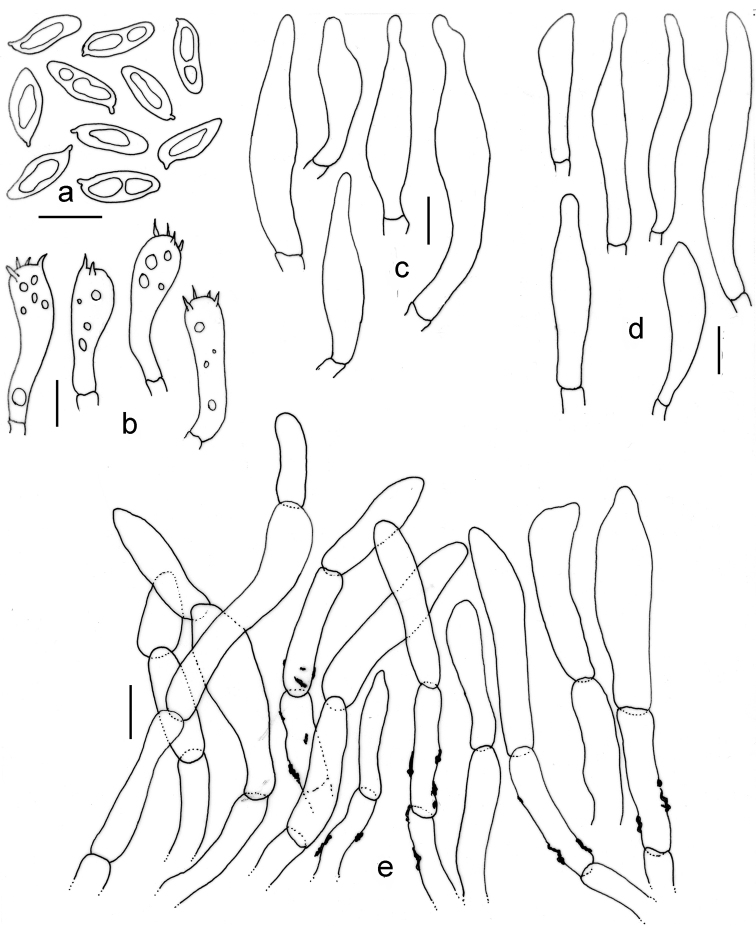
*Tylopilus himalayanus* (DC 17-25, holotype). **a** Basidiospores **b** Basidia **c** Pleurocystidia **d** Cheilocystidia **e** Pileipellis. Scale bars: 10 μm (**a–e**).

##### Specimens examined.

India. Sikkim: Upper Chandmari, 1977 m alt., N27°23'13.7", E88°46'42.9", under *Pinus* sp. in temperate mixed forest, 26 Aug 2017, *D. Chakraborty & K. Das*, DC 17-31 (CAL); Uttarakhand: Champawat district, Abbot mount, 1933 m alt., N29°25.465', E80°06.422', under *Cedrus deodara* in temperate coniferous forest, 18 Jul 2017, A. Ghosh, KD B-03 (CAL).

#### Tylopilus pseudoballoui

Taxon classificationFungiBoletalesBoletaceae

K. Das, D. Chakr & Vizzini sp. nov.

MB823977

Figs 5 , 6

##### Type.

INDIA. Sikkim, South District, Maenam WLS (Maenum 3), 2136 m alt., N27°15'34.7" E88°21'25.7", 23 Aug 2017, *Quercus* spp., *D. Chakraborty & K. Das*, DC 17-30 (CAL 1651)

##### Diagnosis.

Distinct from all allied taxa by sequence data (nrITS and nrLSU) and morphologically by its sticky orange-yellow pileus surface, pale yellow pore surface which turns to pale orange or greyish-orange when bruised and absence of reticulation on stipe surface.

##### Etymology.

referring to its being a look-alike of *T. balloui*, a North American species.

##### Description.

Pileus 60–150 mm diam., initially convex then plano-convex, surface sticky, orange-yellow to brownish-yellow (5B–C8), paler at margin; margin entire, plain, without any sterile flap of tissue. Pore surface pale yellow (3A3), turning pale orange to greyish-orange (6A–B3) on bruising; pores angular, 5–8/mm. Tubes subdecurrent, 6–10 mm long, yellowish-white, brownish on bruising. Stipe 55–110 × 20–40 mm, mostly subclavate, solid, concolorous with pileus; surface pruinose, never reticulate; basal mycelium white. Context 20 mm thick in pileus, chalky white (1A1), unchanging on exposure but turning turquoise grey (24D2–D1) with FeSO_4_ (chalk), pale yellow (4A3) with 5% KOH, yellowish-grey (4B3) in Guaiacol. Pileus surface brownish-red (8C8–7) on bruising, turning dark green to greenish-grey (25E–F3–2) in FeSO4, vivid yellow (3A8) in KOH, unchanging in NH_4_OH. Stipe 55–110 × 20–40 mm, mostly subclavate, solid, concolorous with pileus; surface pruinose, never reticulate; basal mycelium white. Odour pleasant. Taste slightly pungent. Spore print not obtained.

Basidiospores 6.4–**7.4**–9.9 × 3.8–**4.5**–5.7 µm (n = 30, Q = 1.5–**1.73**–2.04), ellipsoidal, thin-walled, smooth under light microscope. Basidia 22– 30 × 8–10 µm, four-spored, clavate. Pleurocystidia 40–54 × 7–10 µm, emergent up to 30 µm, fusoid to ventricose, appendiculate, yellow pigmented or hyaline, mostly with dense globular to oily content. Tube edge fertile; cheilocystidia 33–55 × 7–10 µm, common, clavate to cylindrical, subfusoid to ventricose. Hymenophoral trama divergent, hyphae septate, gelatinous, up to 5 µm wide. Pileipellis an ixocutis up to 150–280 µm thick, composed of subparallel to suberect, somewhat interwoven hyphae; terminal elements 20–70 × 6–11 µm, cylindrical to subcylindrical, sometimes subfusoid, content orange-brown pigmented. Stipitipellis up to 150 µm thick, fertile, composed of basidia and cystidia in several clusters; caulobasidia similar to that of hymenial basidia; caulocystidia 40–76 × 10–12 µm, broadly clavate to subclavate or appendiculate.

**Figure 5.**
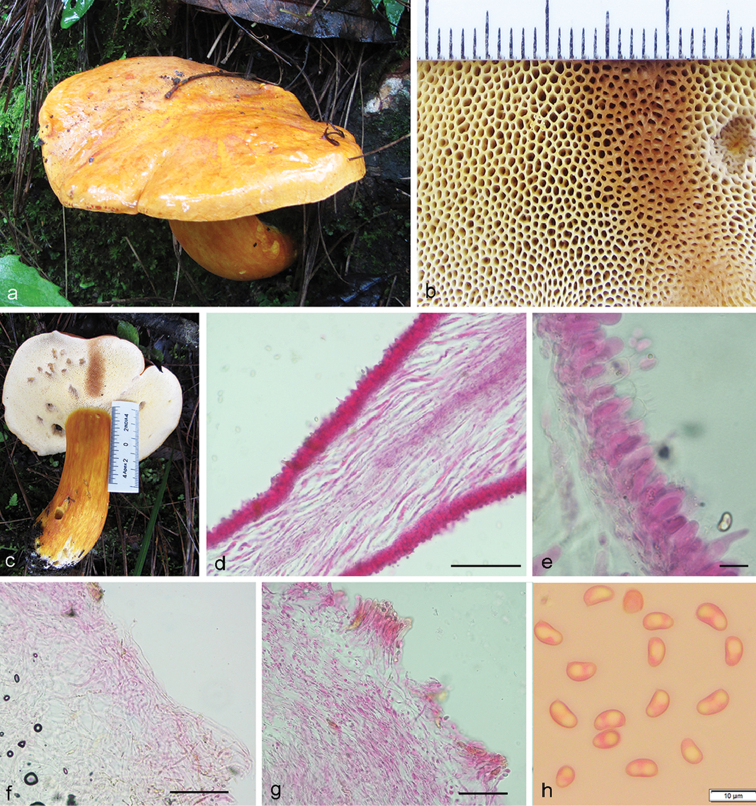
*Tylopilus pseudoballoui* (DC 17-30, holotype). **a, c** Fresh basidiomata in the field **b** Pore surface after bruising **d** Hymenophoral trama **e** Pleurocystidia **f** Pileipellis **g** Stipitipellis **h** Basidiospores. Scale bars: 100 μm (**d, f**); 50 μm (**g**); 10 mm (**e, h**).

**Figure 6.**
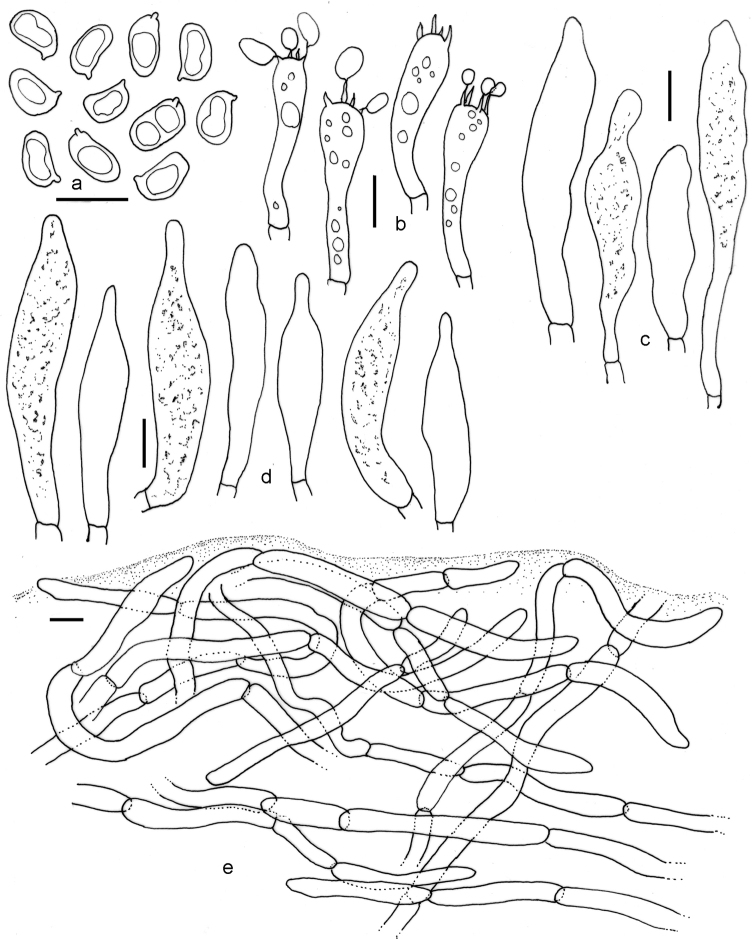
*Tylopilus pseudoballoui* (DC 17-30, holotype). **a** Basidiospores **b** Basidia **c** Caulocystidia **d** Pleurocystidia **e** Pileipellis. Scale bars: 10 μm (**a–e**).

##### Specimens examined.

India. Sikkim: South District, Maenam WLS (Maenam 3), 2136 m alt., N27°15'34.7", E88°21'25.7", 23 Aug 2017, *Quercus* sp., *D. Chakraborty & K. Das*, DC 17-35 (CAL); Uttarakhand: Champawat district, Abbot mount, 1885 m alt., N29°25.466', E80°06.085', 18 July 2017, A. Ghosh, KD B-02 (CAL). Uttarakhand: Pauri district, 1971 m alt., N30°02.874', E79°08.221', 4 Aug 2017, *K. Das*, KD 17-24 (CAL).

#### Tylopilus neofelleus

Taxon classificationFungiBoletalesBoletaceae

Hongo, J. Jpn. Bot. 42: 154 (1967)

Figs 7 , 8

##### Description.

Pileus 70–120 mm broad, convex to broadly convex; surface distinctly tomentose to subvelvety, dry, subviscid when wet; reddish-brown (8–9F4) when young, rosewood (9C5) to vinaceous-brown (16C5) with maturity, fawn (7E4) towards margin. Pores yellowish-white or cream with a pinkish tinge, orange-grey (6B2) with age; pore 2–3/mm, rounded. Tubes 10–15 mm, adnate-sinuate, white in colour, yellow-brown or orange white with maturity. Stipe 60–100 × 18–22 mm, cylindrical, solid, surface dry, glabrous to subvelutinous, typically distinctly reticulate at apex, reticulation greyish-ruby (12C–D4) to dark ruby (12F5); surface pinkish brown to vinaceous or violaceous, dark brown to reddish-brown with maturity. Context chalky white, but pinkish-brown when exposed. Spore print not obtained.

Basidiospores 10–**11.5**–13.5 × 4–**4.6**–5.2 µm (n = 30, Q = 2.05–**2.48**–2.76), ellipsoid to narrowly subfusoid, inequilateral, smooth, thin-walled. Basidia 30–36 × 10–11 µm, 4-spored, clavate, thin-walled, hyaline or pale grey in KOH. Pleurocystidia 35–66 × 14–24 µm, scattering and numerous, fusoid-ventricose or subclavate, with orange brown contents. Cheilocystidia 33–38 × 9–12 µm, ventricose to fusoid, shorter and smaller than pleurocystidia thin-walled, with orange brown contents. Pileipellis 100–150 µm thick, an ixotrichoderm of suberect, branched, septate hyphae; terminal elements ventricose to fusoid, vaculolated, 28–50 × 12–14 µm, with granular yellowish to brown orange contents in KOH; subterminal elements mostly with incrustations. Stipitipellis 35–65 µm, fertile, composed of basidia and cystidia. Caulocystidia 52–63 × 8–13 µm, fusoid to subfusoid, ventricose to ventricose-rostrate or narrowly cylindrical, content granular. Clamp connection absent in all tissues.

**Figure 7.**
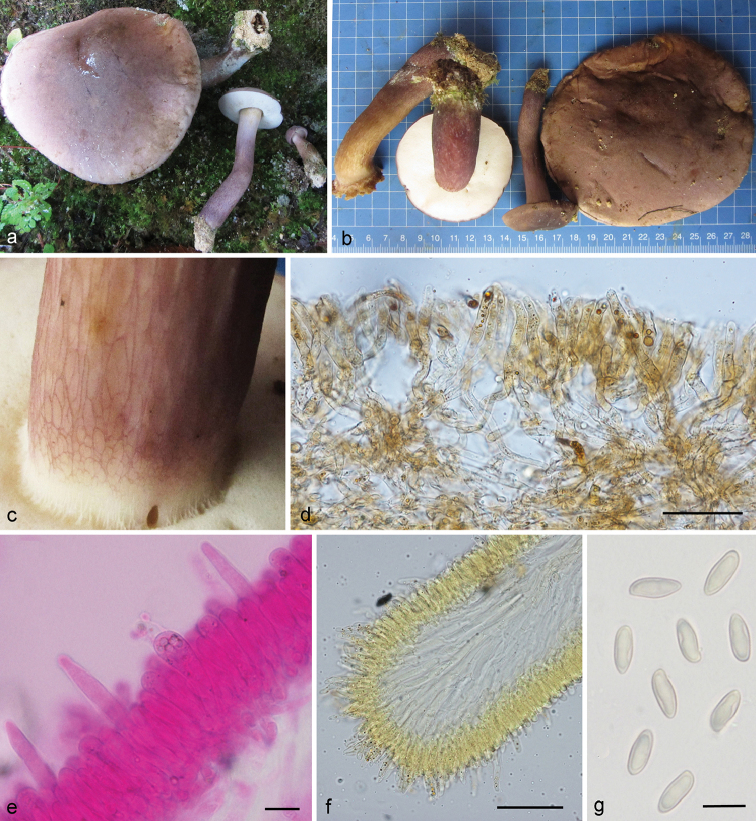
*Tylopilus neofelleus* (DC 16-63). **a, b** Fresh basidiomata in the field and in basecamp **c** Stipe surface with reticulation **d** Pileipellis **e** Hymenial layer showing basidia and pleurocystidia **f** Tube edge **g** Basidiospores. Scale bars: 50 μm (**d, f**); 10 μm (**e, g**).

**Figure 8.**
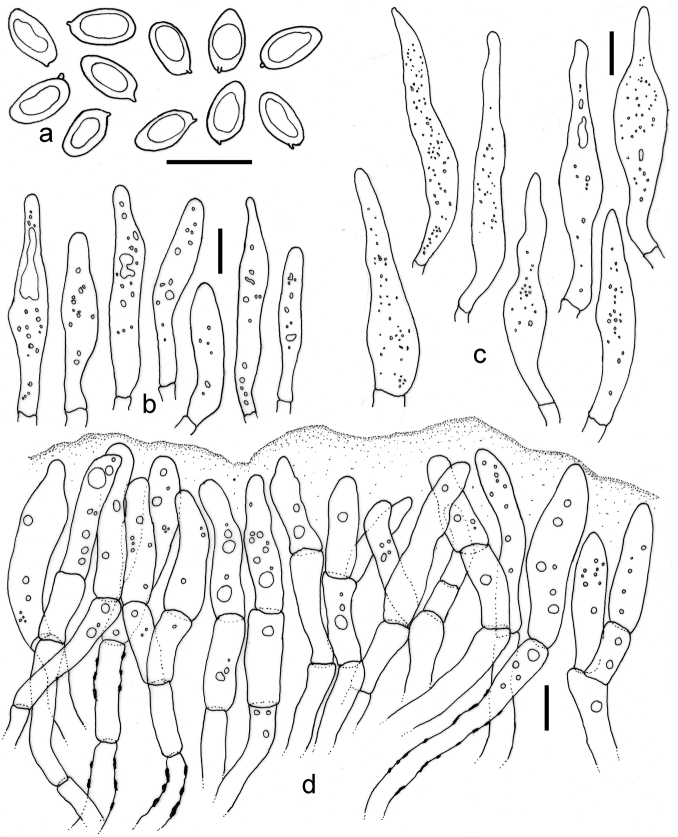
*Tylopilus neofelleus* (DC 16-63). **a** Basidiospores **b** Pleurocystidia **c** Caulocystidia **d** Pileipellis. Scale bars: 10 μm (**a–d**).

##### Habitat.

Under *Castanopsis* sp. in temperate broadleaf forest.

##### Known distribution.

Japan (Kawamura 1954; Hongo 1960; Imazeki et al. 1970, 1988; Takahashi 1986; Gelardi et al. 2015), China (Ying and Zang 1994; Li and Song 2000; Wang et al. 2004; Fu et al. 2006; Wu et al. 2011; Gelardi et al. 2015), Russia (Vasil’jeva 1978) and New Guinea (Hongo 1973).

##### Specimens examined.

India. Sikkim: East district, Fambonglo WLS, 2021 m alt., N27°21'47.5" E88°34'13.2", 26 Aug 2016, *D. Chakraborty & K. Das*, DC 16-63 (CAL); ibid., *D. Chakraborty & K. Das*, DC 16-64 (CAL).

## Discussion

Our first novel species in *Tylopilus* s.s. (Wu et al. 2014), i.e. *T. himalayanus*, is featured by its brown, reddish-brown to purplish-grey, dry pileus, angular pores, stipe usually without reticulum even though sometimes with faintly reticulate apex, but longitudinally striate throughout, white unchanging context on exposure, bitter taste, sterile tube edge and trichodermic structure of pileipellis. Morphologically, *T. rubrobrunneus* Mazzer & A.H. Sm., *T. felleus* (Bull.) P. Karst., *T. neofelleus* Hongo and *T. intermedius* A.H. Sm. & Thiers resemble *T. himalayanus*. *Tylopilus rubrobrunneus* (originally reported from North America) differs from this species by its olive tinge on stipe surface, pileus surface with vinaceous tinges, rounded pores, negative colour reaction with KOH or NH_4_OH on context (Mazzer and Smith 1967, Smith and Thiers 1971, Grund and Harrison 1976, Both 1993, Bessette et al. 2010, 2016). Similarly, *T. intermedius* differs from the present Indian species by possessing a distinctively whitish pileus that stains pinkish buff to brown with age and context (pileus) that turns pinkish with FeSO_4_, but remains unchanged with KOH (Smith and Thiers 1971, Both 1993, Bessette et al. 2010, 2016). Some other members of this genus, such as *T. felleus* (originally described from Europe and known from India as well without checking its conspecificity through phylogeny), *T. neofellus* (originally reported from Japan but reported here for the first time from India), *T. plumbeoviolaceus* Snell & Dick (originally reported from North America but also known from this country without verifying its conspecificity through phylogeny) and *T. violatinctus* T.J. Baroni & Both (originally reported from North America), can also be separated from *T. himalayanus* morphologically: *T. felleus* has brownish pileus and distinctively reticulate stipe (Lannoy and Estadès 2001, Muñoz 2005); *T. neofellus* and *T. plumbeoviolaceus* possess reddish-brown to violaceous-brown pileus and reticulate purplish-violaceous stipe (Snell and Dick 1941, Smith and Thiers 1971, Grund and Harrison 1976, Both 1993, Lakhanpal 1996, Bessette et al. 2010, 2016, Gelardi et al. 2015); *T. violatinctus* is easily distinguished by the more brightly coloured, bluish-violet to lilac-lavender or purple-greyish pileus, bruising dark rusty-violet when handled, the stipe turning yellowish on bruising, pileus surface and context staining yellowish-brown and negative to pinkish-brown with KOH, respectively, small basidiospores [(5.6–)7–9(–10) × 3–4 µm] and the growth in mixed woodlands possibly with *Quercus*, *Fagus* or *Picea*, in any case not in association pine or cedar trees (Baroni and Both 1998, Ortiz-Santana et al. 2007, Bessette et al. 2010).

*Tylopilus balloui*, as currently circumscribed (pileus orange-yellow and short elliptical, pale-coloured basidiospores), represents a species complex rather than a single species, based on morphological data (Watling and Gregory 1988, Li and Watling 1999, Watling and Lee 1999, Watling and Li 1999, Watling 2001a, b, Watling et al. 2006) and molecular phylogenetic inference (Halling et al. 2008, Osmundson and Halling 2010, Magnago et al. 2017 and our analyses, Figs 1–2, Suppl. materials 1–2). Due to its spore shape, *Boletus balloui* Peck was previously considered as a *Gyrodon* (Snell 1941), a *Gyroporus* (Horak 2011) or a *Rubinoboletus* (Heinemann & Rammeloo 1983), but recent molecular studies (Halling et al. 2008; Osmundson and Halling 2010, Trappe et al. 2013, Wu et al. 2014, Magnago et al. 2017, Orihara and Smith 2017) confirmed its position in *Tylopilus* s.s. The true *T. balloui* has to be restricted only to the North American collections (Halling et al. 2008, Osmundson and Halling 2010). Our second novel species, i.e. *T. pseudoballoui*, a distinct species in this complex, is characterised by robust basidiomata with sticky orange to brownish-yellow pileus; pale yellow pore surface that turns to greyish-orange to orange on bruising, angular pores; concolorous stipe, pruinose, never reticulate; context white, unchanging on bruising or when exposed; pileipellis an ixocutis with somewhat interwoven hyphae; possessing two types of hymenial cystidia (hyaline and pigmented with yellowish globular to oily content); and occurrence under *Quercus* spp. In the field, the present species can be confused with its closest look-alike *T. balloui* (Peck) Singer which was originally reported from North America. However, *T. balloui* differs from the Indian species by possessing a dry pileus surface (sticky in *T. pseudoballoui*), white to dingy-white pores and a context turning pinkish-tan on exposure. (Smith and Thiers 1971, Wolfe 1981, Both 1993, Bessette et al. 2010, 2016, Osmundson and Halling 2010). *Tylopilus oradivensis* Osmundson & Halling, described recently from Costa Rica, possesses longer spores, (7.6–)8.2–12(–13.6) × (2.6–)3-4(–4.4) µm and a dry pileus surface, (Osmundson and Halling 2010). *Tylopilus leucomycelinus* (Singer & M.H. Ivory) R. Flores & Simonini from Honduras and Guatemala, has a dry, fibrillose to squamulose pileus surface, abundant white basal mycelium, smaller spores, (5.8–)6.1–6.7(–7.3) × (3.4–)3.8–4.3(–4.9) µm and is associated with *Pinus* spp. (Singer et al. 1983, Flores Arzù and Simonini 2000). Moreover, our twofold phylogenetic analysis clearly separates *T. pseudoballoui* (also known from Japan as clearly indicated in our Figs 2 and Suppl. material 2). Boletus balloui var. fuscatus Corner from Malaysia, is morphologically similar to the Indian collection but the former differs by its narrower stipe (width 7–24 mm at apex, 3-12 mm at base), fawn-ochraceus pileus surface, dull purple brown pore surface on bruising, vinaceous to dull purple context on exposure (context unchanging in *T. pseudoballouii*), sterile stipitipellis and low land distribution (1300 mm alt.) (Corner 1972). *Tylopilus viscidulus* (Pat. & Baker) Watl. & Lee also known from Malaysia, differs from the Indian species by its pale cream coloured pileus and stipe, smaller size of basidiomata (pileus 25–40 mm diam. and stipe 20–35 × 8–15 mm), pale brown colour of context on exposure and presence of lageniform cystidia (Patouillard and Baker 1918, Watling and Lee 1999). Finally, Rubinoboletus balloui var. viscidus T.H. Li & Watling from Australia is distinguished by a smaller pileus (up to 70 mm broad), context turning pinkish on cutting and longer spores, 7.5–11.0 × 4.0–4.8 µm (Li and Watling 1999, Watling and Li 1999).

The combination of morphological features in Indian collections of *T. neofelleus* and two-fold phylogeny (MG777529, MG777525 in Figs 1 and Suppl. material 1; MG777524, MG777523 in Figs 2 and Suppl. material 2) attest the conspecificity of these collections with their Chinese or Japanese counterparts. *Tylopilus neofelleus* (= *T. microsporus* S.Z. Fu, Q.B. Wang & Y.J. Yao fide Gelardi et al. 2015) is closely related to *T. felleus* (Bull.) P. Karst. and *T. plumbeoviolaceus* (Snell & E.A. Dick) Snell & E.A. Dick. However, *T. felleus* (originally reported from Europe, Munoz 2005) has a brown pileus with olive-grey colour and distinctively brown reticulation on its yellowish stipe-surface, while *T. plumbeoviolaceus* (originally reported from North America) has a deep violet-purplish, then purple-brown to dull cinnamon-brown pileus. Micromorphologically, basidiospores of *T. plumbeoviolaceus* are distinctively longer [10–13(–14) × 3–4(–5.5) µm, than those of *T. neofelleus* (Smith & Thiers 1971, Bessette et al. 2000, 2006, Gelardi et al. 2015). *Tylopilus plumbeoviolaceoides* T.H. Li, B. Song & Y.H. Shen, described from China, differs in the darkly coloured pileus and stipe ranging from dark violaceous to brown-vinaceous, the context turning pinkish to purplish when cut, and usually longer and somewhat narrower spores [(7.5–) 8.5–10.5(–12) × (2.5–)3.0–3.8(–4.2) µm] (Li 2011, Gelardi et al. 2015). Finally, *T. himalayanus* is distinct from *T. neofelleus* by the absence of purplish-violaceous tinges on the stipe surface and of a well-developed reticulum.

## Supplementary Material

XML Treatment for Tylopilus himalayanus

XML Treatment for Tylopilus pseudoballoui

XML Treatment for Tylopilus neofelleus

## Supplementary materials

Supplementary material 1 Figure S1 Data type: (measurement/occurence/multimedia/etc.) Explanation note: nrLSU based Bayesian phylogram inferred from MrBayes under TIMef model of nucleotide evolution. Two species of *Xanthoconium* (*X. sinense* and *X. purpureum*) were used as outgroup taxa. Posterior probabilities values (BPP) are indicated above or below the respective branches. New taxa or new record for Indian mycobiota are shown in bold and red in the phylogram. This is an open access article distributed under the terms of the Creative Commons Attribution License (CC BY 4.0), which permits unrestricted use, distribution, and reproduction in any medium, provided the original author and source are credited.Dyutiparna Chakraborty, Alfredo Vizzini, Kanad Das

Supplementary material 2 Figure S2 Data type: (measurement/occurence/multimedia/etc.) Explanation note: nrITS based Bayesian phylogram inferred from MrBayes under TrNef+G model of nucleotide evolution. *Gyroporus castaneus* was used as outgroup species. Posterior probabilities values (BPP) are indicated above or below the respective branches. New taxa or new record for Indian mycobiota are shown in bold and red in the phylogram. This is an open access article distributed under the terms of the Creative Commons Attribution License (CC BY 4.0), which permits unrestricted use, distribution, and reproduction in any medium, provided the original author and source are credited.Dyutiparna Chakraborty, Alfredo Vizzini, Kanad Das
