# Supplementary figures and images for: Two new species and one new record of the genus Tylopilus (Boletaceae) from Indian Himalaya with morphological details and phylogenetic estimations
Source: MycoKeys. 2018 Apr 13;(33):103–24. doi: 10.3897/mycokeys.33.23703 (PMC5911683; doi:10.3897/mycokeys.33.23703)

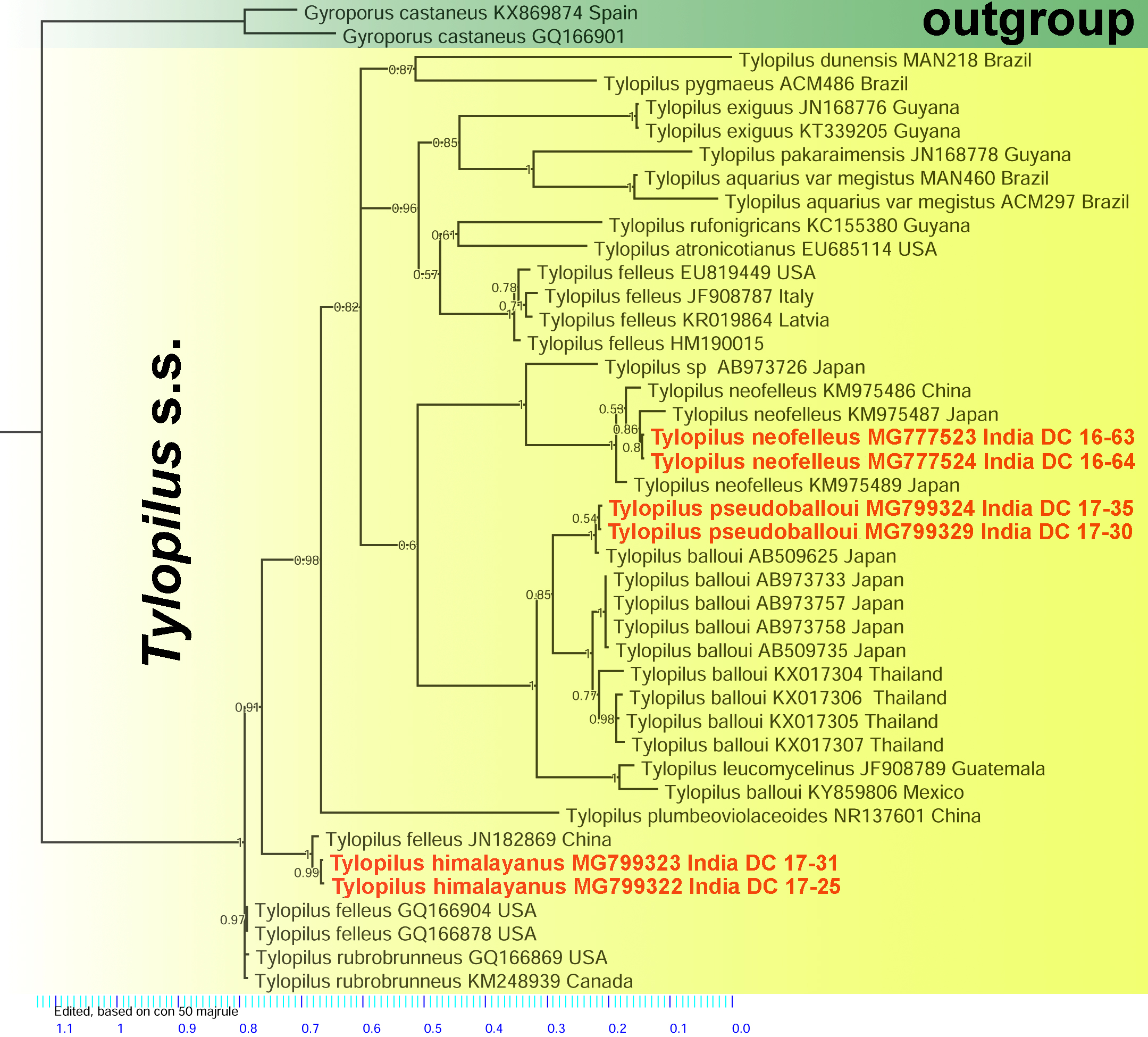

Supplement: Supplementary material 1 — Figure S1 [file mycokeys-33-103-s002.jpg]
